# Effects of Copper Addition on Copper Resistance, Antibiotic Resistance Genes, and *intl1* during Swine Manure Composting

**DOI:** 10.3389/fmicb.2017.00344

**Published:** 2017-03-03

**Authors:** Yanan Yin, Jie Gu, Xiaojuan Wang, Wen Song, Kaiyu Zhang, Wei Sun, Xin Zhang, Yajun Zhang, Haichao Li

**Affiliations:** ^1^College of Resources and Environmental Sciences, Northwest A&F UniversityYangling, China; ^2^College of Science, Northwest A&F UniversityYangling, China

**Keywords:** antibiotic resistance gene, bacterial community, composting, copper resistance gene, Cu

## Abstract

Copper is one of the most abundant heavy metals present in swine manure. In this study, a laboratory-scale aerobic composting system was amended with Cu at three levels (0, 200, and 2000 mg kg^-1^, i.e., control, Cu200, and Cu2000 treatments, respectively) to determine its effect on the fate of copper resistance genes [copper resistance genes (CRGs): *pcoA, cusA, copA*, and *tcrB*], antibiotic resistance genes [antibiotic resistance genes (ARGs): *erm*(A) and *erm*(B)], and *intl1*. The results showed that the absolute abundances of *pcoA, tcrB, erm*(A), *erm*(B), and *intl1* were reduced, whereas those of *copA* and *cusA* increased after swine manure composting. Redundancy analysis showed that temperature significantly affected the variations in CRGs, ARGs, and *intl1*. The decreases in CRGs, ARGs, and *intI1* were positively correlated with the exchangeable Cu levels. The bacterial community could be grouped according to the composting time under different treatments, where the high concentration of copper had a more persistent effect on the bacterial community. Network analysis determined that the co-occurrence of CRGs, ARGs, and *intI1*, and the bacterial community were the main contributors to the changes in CRGs, ARG, and *intl1*. Thus, temperature, copper, and changes in the bacterial community composition had important effects on the variations in CRGs, ARGs, and *intl1* during manure composting in the presence of added copper.

## Introduction

Poultry and livestock feeds are frequently supplemented with trace elements such as copper to promote optimum growth due to their antimicrobial properties (Xiong et al., 2010). However, poultry and livestock absorb these additives at low rates and they excrete up to 95% in their dung and urine, which often contain high concentrations of heavy metals such as copper (Xiong et al., 2010). High concentrations of copper have been found in manure (22–3388 mg kg^-1^; Ji et al., 2012), and large amounts of copper may enter the environment via the application of manure. Thus, the ecological environment may be damaged by copper, which could perturb the structure and function of the microbial community.

Composting is a common and effective approach for manure treatment before its application to land. In the composting process, microbes play a key role in the biotransformation (i.e., biosynthesis or biodegradation) of compost materiel (Guo et al., 2012). In addition, copper can impose selective pressures on the bacterial community’s tolerance of copper during composting (Li et al., 2014), which may be attributable to bacteria carrying copper resistance gene (CRGs) in the bacterial community. CRGs may reflect the actual responses of bacteria to the selective pressure imposed by copper, but the effects of copper on the evolution of CRGs during swine composting are still unclear. The main mechanisms that mediate copper resistance in microorganisms include the following: (1) the efflux ATPase pump encoded by *copA* can extrude copper ions from the cytoplasm into the periplasmic space (Rensing and Grass, 2003); (2) the cus system where the *cusA* gene encodes a resistance nodulation cell protein with an antiport system (Outten et al., 2001); (3) the pco system for Cu resistance encodes a multicopper oxidase protein, which is responsible for the oxidation of copper (I) to copper (II) in the periplasmic space (Brown et al., 1997); (4) the cue system is the main mechanism responsible for copper resistance in *E. coli*, where *cueO* encodes a periplasmic multicopper oxidase (Outten et al., 2001; Rensing and Grass, 2003); and (5) *tcrB* in *Enterococcus faecium* belongs to the CPX-type ATPase family of heavy metal transporters (Henrik and Frankm, 2002). The bacteria that carry these genes may be transferred from human and animal sources into the environment, thereby leading to disease. For example, Mourão et al. (2015a) found that *pcoA*-*pcoD* were chromosomally located in *Salmonella enterica* serotype 4,[5],12:i:, which is a new epidemic multidrug-resistant serotype in Europe that is associated with human infections. In *E. faecium, tcrB* belongs to the CPx-type ATPase family, and human homologs of the human CPx-type ATPase are related to Menkes and Wilson disease (Trenor et al., 1994).

Recently, pollution-induced community tolerance (PICT) was elucidated based on the rate of microbial metabolism and several researchers have represented the copper resistance of the microbial community as PICT (Fernández-Calviño and Bååth, 2013; Li et al., 2014). However, this has only been shown for the culturable fraction of bacteria (Huysman et al., 1994). Screening resistant bacteria by culture only samples microbes that are culturable and that can express their CRGs under these conditions. Recently, culture-independent methods have been investigated for quantifying the abundances of CRGs using quantitative PCR (qPCR; Besaury et al., 2013; Xiong et al., 2015; Zhang et al., 2016a). In addition, heavy metal and antibiotic resistance are frequently linked on the same plasmid (Baker-Austin et al., 2006; Laffite et al., 2016), and increased mobilization under metal-selective conditions might increase the risk of antibiotic resistance genes (ARGs) spreading via horizontal gene transfer to bacteria that cause human infections (Zhu et al., 2013; Martínez et al., 2014). The integron platforms are defective for self-transposition, but they are often associated with transposons and/or conjugative plasmids, which can serve as vehicles for the intra- and interspecies transmission of genetic material (Mazel, 2006). Gillings et al. (2015) proposed that the class 1 integron-integrase gene (*intI1*) can be used as a generic marker for anthropogenic pollutants. However, Pal et al. (2015) showed that no CRGs co-occur with *intI1* or any other ARGs on plasmids based on large-scale genomic analyses of bacterial genomes and plasmids. Thus, it is necessary to obtain a better understanding of the changes in CRGs as well as the relationship between CRGs and the ARGs and *intl1* during swine manure composting in the presence of added copper.

In the present study, we investigated the changes in CRGs, ARGs, and *intl1* using qPCR, and we employed 16S rRNA high-throughput sequencing to determine the changes in the bacterial community. The results of this study should help to determine the effects of copper exposure on CRGs, ARGs, *intl1*, and the bacterial community during the manure composting process, thereby providing a better understanding of how and why the abundances of CRGs/ARGs change during composting under copper exposure.

## Materials and Methods

### Experimental Setup

The manure was collected from a medium-sized swine farm in Yangling, Shaanxi, China (i.e., producing swines > 500 head/year), where the diet comprised corn, bran, and soybean meal. The pre-mix feed included tetracycline, macrolide, CuSO_4_, and ZnSO_4_. In order to replicate the actual responses of resistance genes at different copper concentrations, the feed given to swine supplemented with trace elements and antibiotics was stopped 2 weeks before collecting the manure. The fresh manure was mixed well, air dried to obtain a water content <30%, crushed, and sieved through a 5-mm mesh. Wheat straw was cut into pieces that measured <1 cm long. The swine manure had an organic nitrogen content of 26 g kg^-1^, organic carbon content of 380.2 g kg^-1^, pH of 7.6, and total copper content of 121 mg kg^-1^. The wheat straw had an organic nitrogen content of 6.5 g kg^-1^ and an organic carbon content of 496.3 g kg^-1^.

The compost reactors comprised 54 identical 500 mL plastic containers, where 150 g dry weight (DW) of the raw material was placed in each container. The swine manure and wheat straw were mixed (3:1 DW) to prepare the compost mixture. The composting experiment was designed as described by Li et al. (2014) and determined based on the Cu residue levels in swine manure (Xiong et al., 2010). A stock solution of Cu was prepared and the diluted Cu solution was then mixed thoroughly with each sample to spike them with additional Cu concentrations of 200 and 2000 mg Cu kg^-1^ (DW), i.e., Cu200 and Cu2000, respectively. A similar volume of sterilized distilled water was added to the control (without the addition of CuSO_4_). Before use in the composting experiment, CuSO_4_ was stabilized for 6 h in swine manure. The moisture content was adjusted to 55%. To provide the optimal conditions for composting, the temperature was artificially controlled in an incubator in the following four phases: mesophilic phase (20–55°C, 0–5 days), thermophilic phase (55°C, 6–10 days; 55–50°C, 11–13 days; 50°C, 14–16 days), cooling phase (50–40°C, 17–21 days), and maturity (decreased to 20°C, 22–35 days), as described previously (Li et al., 2014). The composts were turned fully and mixed every 2 days during the composting process.

Three plastic containers for each treatment were then sampled as triplicates on days 0, 2, 7, 14, 21, and 35, and these samples were mixed well to form a test subsample. The sample was divided into two parts, where one was used to determine the physical and chemical properties, and the second was freeze-dried using a vacuum freeze dryer (Songyuan, China), milled to 1 mm by an ultra-centrifugal mill (Retsch Z200, Germany), and stored at –80°C.

### Chemical Analysis

The moisture contents of fresh samples were measured by drying at 105°C for 24 h. The pH values were determined based on fresh samples in water suspension at 1:10 (w/w) using a Thermo Orion 3-star pH-meter (San Diego, CA, USA). NH_4_^+^ and NO_3_^-^ were extracted with 2 M KCl and determined by flow injection analysis (Systea, Italy).

Cu was sequentially extracted, as described by Tessier et al. (1979), where the extracts were then analyzed using a Model Z-2000 Series Polarized Zeeman atomic absorption spectrophotometer (Hitachi, Japan). For the data shown in Supplementary Figure S1 and Miaomiao et al. (2009), exchangeable Cu is defined as the bioavailable Cu in swine manure compost.

### DNA Extraction, qPCR, and Sequencing Analysis

DNA was extracted from the freeze-dried compost samples using a Fast DNA Kit for Soil (MP Biomedicals, USA) according to the manufacturer’s instructions. Five CRGs (*copA, cusA, cusO, pcoA*, and *tcrB*), five macrolide resistance genes [*erm*(A), *erm*(B), *erm*(C), *erm*(T), and *erm*(X)], and the integrase gene of class 1 integrons (*intI1*) were determined by standard PCR. In addition, four CRGs (*copA, cusA, pcoA*, and *tcrB*), two macrolide resistance genes [*erm*(A) and *erm*(B)], and the integrase gene of class 1 integrons (*intI1*) were detected by standard PCR. These genes were quantified by real-time qPCR, where the conditions and primer sequences are described in the Supporting Information (Supplementary Material S1 and Tables S1a,b). The samples were quantified by qPCR using the Bio-Rad CFX connect^TM^ Real-Time PCR Detection System (Bio-Rad). The absolute abundances of the CRGs and ARGs were expressed as copies g^-1^ of dry compost. The relative abundances of the CRGs and ARGs were calculated as: copy number of a CRG or ARG/copy number of 16S rRNA.

The V4 region of the 16S rRNA gene was analyzed by high-throughput sequencing using the Illumina HiSeq platform at Novogene (Beijing, China). The raw data were analyzed using QIIME software and the UPARSE pipeline (Caporaso et al., 2010). The UPARSE pipeline was employed for taxonomic assignment based on ≥97% similarity. Taxonomic classification was performed using the RDP classifier (Wang et al., 2007). All the raw sequence data have been deposited in the NCBI SRA database (Accession number: SRP093470).

### Statistical Analyses

All of the statistical analyses were performed using SPSS 19.0 (SPSS Inc., USA). Significant differences in the relative abundances of CRGs and ARGs (at *P* < 0.05) between treatments were detected using a one-way ANOVA/LSD test. Principal coordinates analysis (PCoA) was conducted using R (Version 2.15.3). Network analysis based on Spearman’s rank analysis was performed using the relative abundances of CRGs, ARGs, the bacterial community (based on OTU), and *intI1* with the Gephi platform. Redundancy analysis was conducted with CANOCO 4.5.

## Results and Discussion

### Changes in Exchangeable Cu Content and the Key Parameters during Composting

As shown in Supplementary Table S2, the pH of the control increased gradually during the first week and the maximum value (pH 9.14) was reached on day 7. The pH then declined to 8.51 in the final compost. The increase in the pH during the initial stage of composting was due mainly to the release of ammonia and the degradation of organic acids (Li et al., 2014), whereas the decrease in the pH during the later phase was probably caused by the synthesis of phenolic compounds, or the nitrifying process (Zhang et al., 2015). In addition, the pH of Cu2000 was slightly lower than that of the control and Cu200 after 21 days, which may have been related to the adverse effects of Cu on the urease activity (Li et al., 2014), thereby inhibiting the release of ammonia. As the pile temperature increased, the NH_4_^+^ value increased during the first 7 days, but then decreased until the end of composting. The NO_3_^-^ value increased on day 2 and decreased gradually during days 7–21, before increasing again from days 21 to 35. However, compared with the control, the NO_3_^-^ values were lower in Cu200 (62.9% lower) and Cu2000 (76.4% lower) on day 35, which may have been related to inhibition of the activity of nitrifying microorganisms by copper.

During composting, the distribution of metals can be influenced by physicochemical changes within the mixture, such as organic matter mineralization, decreases in pH, and the formation of humic substances (Hsu and Lo, 2001; Farrell and Jones, 2009). The exchangeable Cu concentrations with different treatments are shown in Supplementary Table S2, which indicate that the exchangeable Cu concentration was 3.10–129.57 mg kg^-1^ during composting, where there was a decrease from early in the first week, although there was a transient increase in the control during the first 2 days. In the cooling phase, the exchangeable Cu contents remained fairly constant. This indicates that the forms of metals were more stable after composting, as shown in previous studies (Tandy et al., 2009; Li et al., 2014).

### Dynamic Changes in CRGs during Composting

After composting for 35 days, the absolute abundances of *pcoA* and *tcrB* decreased (Supplementary Tables S3a,b), which might have been due to cell death. Burch et al. (2013) found that death of the bacterial host led to reductions in ARGs. In addition, composting provides aerobic conditions, which might have been unfavorable for the maintenance of *pcoA* and *tcrB*, e.g., *pcoA* and *tcrB* may function better under anaerobic conditions than aerobic conditions (Mourão et al., 2015a,b). However, the abundances of *copA* and *cusA* increased during the composting process (Supplementary Figure S2 and Table S3b). Outten et al. (2001) and Toes et al. (2008) found that *copA* and *cusA* were expressed under copper stress in aerobic and anaerobic conditions. Furthermore, the abundance of *copA* was higher than that that of *cusA* at the end of composting, especially in the Cu200 and Cu2000 treatments (Supplementary Tables S3a,b), which indicates that *copA* dominated when copper was added compared with *cusA*. By contrast, Besaury et al. (2014) reported that *cusA* was dominant compared with *copA* in copper-contaminated sediment, where this difference may be explained by variations in the evolution of the bacterial communities in the swine compost and sediment environments.

**FIGURE 1:**
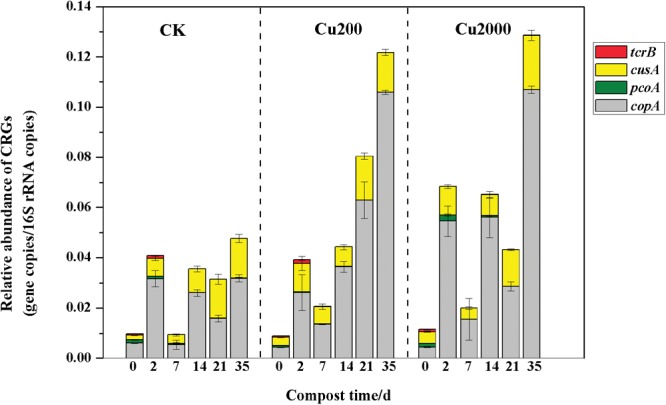
**Changes in the relative abundances of copper resistance genes (CRGs) under different manure composting treatments**.

The variations in the relative abundances of CRGs are shown in **Figure 1**, Cu2000 enhanced the abundance of *pcoA* by 1.7–6.0 times compared with the control during days 2–14 (except on day 7), so it is probable that most of the microbes carrying the *pcoA* gene were enhanced by the high concentration of copper in the early composting phase. However, Cu2000 increased the abundance of *tcrB* by 1.3–8.9 times and 4.7–15.2 times compared with the control and Cu200, respectively, during days 14–35, which indicates that the bacterial hosts carrying *tcrB* may have increased under a high concentration of copper, as reported previously (Amachawadi et al., 2015). In addition, Cu200 and Cu2000 enhanced the abundances of *cusA* (1.4–2.4 times) and *copA* (1.7–2.9 times) compared with the control throughout the entire composting process, but there were no significant differences between Cu200 and Cu2000. This suggests that the abundances of the bacteria carrying *copA and cusA* increased during composting. Based on these results, we conclude that Cu2000 slowed down the loss of CRGs during composting.

### Changes in ARGs and *intI1*

**FIGURE 2:**
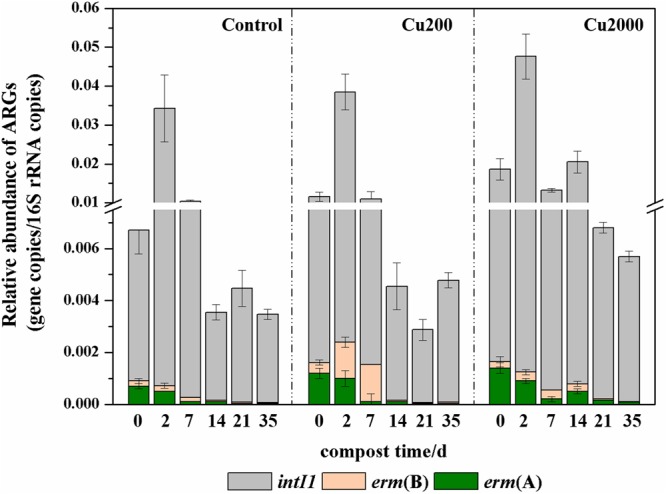
**Changes in the relative abundances of antibiotic resistance genes (ARGs) and *intI1* under different manure composting treatments**.

Copper resistance genes and ARGs are commonly found on plasmids, and copper can potentially co-select for resistance to multiple antibiotics, especially the *erm* macrolide resistance gene (Amachawadi et al., 2013). As shown in Supplementary Figure S3, there were marked decreases in the absolute abundances of *erm*(A) (0.4–1.2 logs) and *erm*(B) (1.2–1.6 logs) after composting for 35 days. *erm*(B) had the highest relative abundance during the composting process (**Figure 2**). The relative abundance of *erm*(B) was significantly higher in Cu200 than that in the control (2.0–8.5 times higher) and in Cu2000 (1.7–4.2 times higher) during days 0–7. The relative abundance of *erm*(B) in Cu2000 was significantly higher than that in the control (1.1–6.1 times) and in Cu200 (1.2–5.9 times) during days 14–35. Compared with the control and Cu 200, Cu2000 enhanced the relative abundance of *erm*(A) by 0.9–2.0 times throughout the entire composting process. These results suggest that the relative abundances of *erm*(A) and *erm*(B) were increased by copper during swine manure composting. Previous studies also found that copper could increase the abundances of macrolide-resistant bacteria in compost (Li et al., 2014) and soil (Fernández-Calviño and Bååth, 2013).

*intI1* genes cannot mobilize and transfer themselves between microbes, but they are often associated with mobile genetic elements such as conjugative plasmids, transposons, and insertion sequences (Wang et al., 2015; Zhang et al., 2016a). In this study, the abundance of *intI1* decreased in all of the treatments (Supplementary Figure S3). Thus, the abundance of *intI1* decreased by 0.7–1.1 logs during the thermophilic phase, but its abundance was stable throughout the subsequent composting period. Cu2000 significantly improved the abundance of *intI1*, and a high absolute abundance of *intI1* in compost can enhance the dissemination of ARGs in the soil environment (Su et al., 2015; Qian et al., 2016a).

### Changes in the Bacterial Community Composition during Composting

This study used 16S rRNA gene sequencing to investigate the responses of the bacterial community to copper stress during composting, where the addition of copper had limited effects on the succession of the bacterial community throughout composting. According to PCoA, PC1, and PC2 accounted for 70.49% of the total variation in the bacterial community (Supplementary Figure S4). The bacterial community structure was separated significantly during different composting periods, thereby indicating that the composting period played an important role in shaping the microbial community, as also shown in previous studies (Qian et al., 2016a; Zhang et al., 2016b). However, Cu2000 was most distinct from the control beyond day 2, and it tended toward the days 0–2 clusters (Supplementary Figure S4). These results demonstrate that higher concentrations of copper had more persistent effects on the bacterial community (Liu et al., 2015; Sun et al., 2016).

**FIGURE 3:**
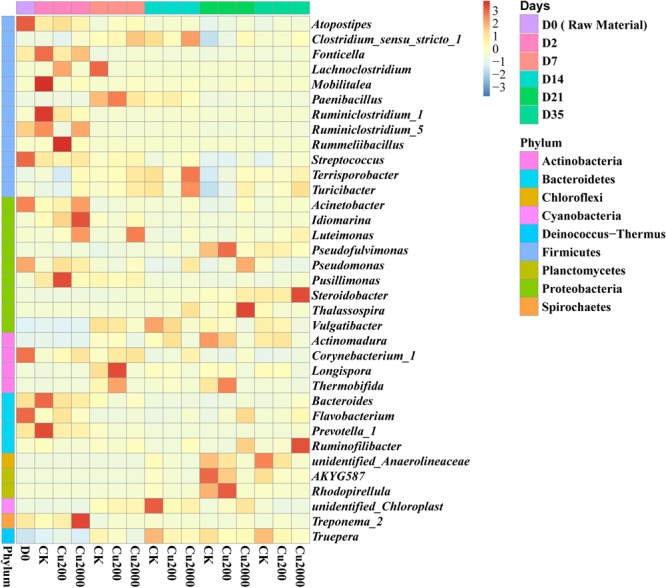
**Heatmap of the top 35 genera identified in compost supplemented with 0, 200, and 2000 mg kg^-1^ copper**.

Among the 34 bacterial phyla identified, the most frequently detected were Firmicutes (17.6–47.0% in all composting treatments), Proteobacteria (16.2–39.2%), Actinobacteria (6.2–23.2%), and Bacteroidetes (2.8–20.7%), which accounted for 68.1.4–94.5% of the total bacteria (Supplementary Figure S5). Firmicutes was the dominant phyla in the mesophilic and thermophilic phases (Supplementary Figure S5). The genus *Clostridium_sensu_stricto_1* was the main contributor to the changes in Firmicutes, and *Clostridium _sensu_stricto_1* strains, which are widely distributed in soil, composts, and the gastrointestinal tracts of animals and humans (Rainisalo et al., 2011), were enhanced in Cu2000 after day 7 (**Figure 3**). Reed et al. (2013) found that *Clostridium* spp. accelerated the utilization of cellulose. Actinobacteria and Proteobacteria were dominant in the cooling and maturation phases. The abundance of Actinobacteria is considered a marker of compost maturity (Wang et al., 2013). Cu2000 decreased the abundance of these bacteria by 38.4% compared with the control on day 35, and the decreased abundance of this phylum after copper addition indicated the lower maturity of the compost obtained. Actinobacteria and Proteobacteria are important hosts of ARGs (Heuer et al., 2011; Stegmann et al., 2015) and previous studies have detected significant positive correlations between them and ARGs (Su et al., 2015; Zhang et al., 2016b). Compared with the control, Proteobacteria were enriched in Cu 2000 throughout composting (Supplementary Figure S5), whereas Actinobacteria were only enriched in Cu 200 and Cu2000 during the mesophilic stage. *Pseudomonas* and *Corynebacterium_1* were the dominant Proteobacteria and Actinobacteria in the raw material (**Figure 3**), and they declined in all treatments by the end of composting. However, compared with the control, they were enriched in Cu2000 during composting. *Pseudomonas* species are highly resistant to and tolerant of copper (Liu et al., 2015), which may be attributed to the presence of the operon *cop* regulating the copper concentration in the cell as well as their ability to produce a large quantity of exopolymer to reduce the toxicity of copper in the soil (Kunito et al., 2001; Andreazza et al., 2012). The genus *Corynebacterium* includes potential human pathogens (Mohammadi et al., 2013), and Wang et al. (2016) claimed that *Corynebacterium* has a positive correlation with *pco* genes. In addition, *Bacteroides* were enriched in Cu200 and Cu2000 after 14 days (**Figure 3**). Liu and Pop (2009) demonstrated that macrolide resistance genes are often present in *Bacteroides*.

### Relationships among Environmental Factors, CRGs, ARGs, *intI1*, and the Bacterial Community

**FIGURE 4:**
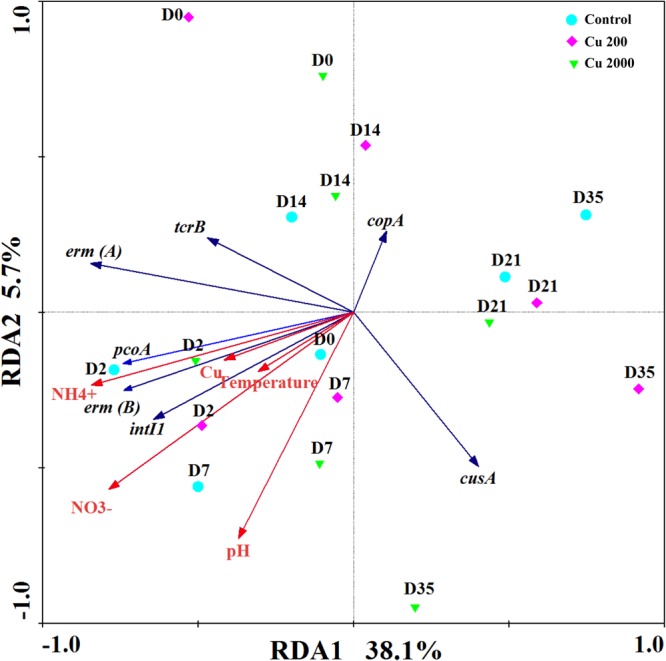
**Redundancy analysis results illustrating the relationships between environmental factors, CRGs, *intI1*, and ARGs during manure composting in the control **(A)**, Cu200 **(B)**, and Cu2000 (C)**. Green triangles, purple diamonds, and blue circles represent samples from **(A–C)**, respectively.

We conducted redundancy analyses to investigate the relationships among environmental factors, CRGs, and ARGs (**Figure 4**), where the results showed that the selected variables accounted for 43.8% of the total variation in the changes in the CRGs and ARGs quantified in this study (*P* = 0.002). Among the environmental factors considered in this study, NH_4_^+^ and NO_3_^-^ significantly explained the variations in the distributions of CRGs, ARGs, and *intl1*. It is possible that the hosts of these genes may have been influenced by the NH_4_^+^ and NO_3_^-^ contents during composting. In addition, temperature significantly explained the decreases in CRGs, ARGs, and *intl1*. A previous study showed that normal thermophilic and continuous thermophilic composting can decrease the abundances of most ARGs and *intl1* (Qian et al., 2016b). Thus, temperature could still reduce the abundances of CRGs, ARGs, and *intl1* under the pressure of a high concentration of Cu. Furthermore, exchangeable copper was positively correlated with *pcoA, tcrB, erm*(A), *erm*(B), and *intl1* (Supplementary Table S4), which indicates that copper supplementation may affect the abundance of bacteria that carry these genes. Heavy metals will be more persistent if they are present inside living bacteria (Ji et al., 2012). Li et al. (2014) claimed that a high concentration of copper increased the microbial PICT to copper and tylosin during swine manure composting.

**FIGURE 5:**
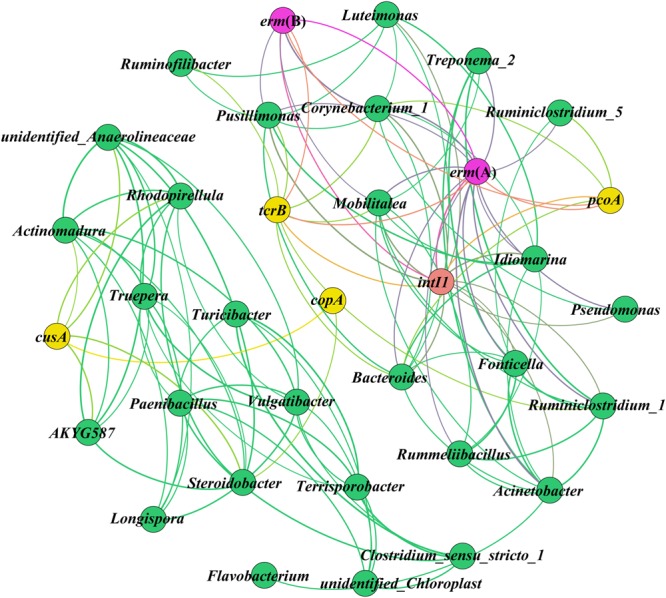
**Network analysis showing the co-occurrence of ARGs, CRGs, and *intl1* as well as their potential host bacteria.** A connection represents a significant positive correlation (*p* < 0.05) according to Spearman’s rank analysis. The size of each node is proportional to the number of connections, i.e., the degree (details are given in the Supplementary Material S2).

New insights into the potential hosts and resistance genes present in environmental samples can be obtained by network analysis (Li et al., 2015; Zhang et al., 2016a). In this study, the CRGs, ARGs, and *intl1* had significant (*p* < 0.05) positive correlations with the same bacterial taxa (**Figure 5**), and it has been demonstrated that these genes posses the same potential host bacteria according to network analysis (Zhang et al., 2016a). The genus *Steroidobacter* may be the potential host bacteria of *copA* and *cusA*, and the increases in these CRGs may have been due to the presence of *Steroidobacter* during composting. The abundance of *Steroidobacter* increased as composting continued, especially in the Cu200 and Cu2000 treatments (**Figure 3**). The co-occurrence of *pcoA, erm*(A), and *intl1* was detected in *Bacteroides* and *Corynebacterium_1*, and the decreases in the abundances of *Bacteroides* and *Corynebacterium_1* during composting may explain the declines in *erm*(A), *pcoA*, and *intl1*. Nesvera et al. (1998) and Nandi et al. (2004) also found *intl1* in Gram-positive bacteria, including *Corynebacterium*. Whittle et al. (2002) demonstrated that *Bacteroides* can acquire resistance genes and transfer themselves and other elements into distantly related bacteria via conjugative transposons. *Corynebacterium_1*, a potential human pathogen (Mohammadi et al., 2013), mainly carries *tcrB* and *erm*(B). Forsberg et al. (2014) and Zhang et al. (2016a) showed that diverse and abundant ARGs are harbored by environmental bacteria and pathogens. In addition, *tcrB* is considered to be present on a conjugative plasmid and the co-localization of *tcrB* with *erm*(B) was demonstrated in a previous study (Amachawadi et al., 2013). Surprisingly, *pcoA*, which is common on plasmids (Besaury et al., 2014), co-occurred with *erm*(A), and *intl1*. This finding is not consistent with the results obtained Pal et al. (2015), who found that no CRGs (including *pcoA*) co-occurred with any ARGs on plasmids, whereas CRGs were connected with many ARGs in the genome. This may be explained by the high intensity of Cu exposure in the swine manure compost, which could differ from that found in other environment samples. Mourão et al. (2015a) found that *pcoA* were chromosomally located in *Salmonella enterica* serotype 4,[5],12:i:, but we did not find that *pcoA* co-occurred with ARGs in *Salmonella*. Thus, the co-occurrence relationships determined by network analysis in the present study need to be validated further using other approaches.

## Conclusion

This is the first study to investigate the fate of CRGs (*pcoA, copA, cusA*, and *tcrB*), ARGs [*erm*(A) and *erm*(B)], and *intI1* during the composting of swine manure with added copper. The abundances of *pcoA, tcrB, erm*(A), *erm*(B), and *intI1* decreased significantly, whereas those of *copA* and *cusA* increased significantly. The high concentration of copper slowed down the dissipation of CRGs, ARGs, and *intI1* during composting. The composting period affected the succession of the bacterial community more greatly than the presence of copper, but a high concentration of Cu had more persistent effects on the bacterial community composition. The succession of the bacterial community, copper, and temperature are important factors that affect the fate of CRGs, ARGs, and *intI1* during manure composting. In addition, we surprisingly detected the co-occurrence of CRGs, ARGs, and *intI1* under copper exposure during swine manure composting, which should be validated using other approaches.

## Author Contributions

YY and JG designed experiments; YY, WSo, XZ, HL, and YZ carried out experiments; YY, XW, WSu, and KZ analyzed experimental results. YY wrote the manuscript.

## Conflict of Interest Statement

The authors declare that the research was conducted in the absence of any commercial or financial relationships that could be construed as a potential conflict of interest.
