# Supplementary material for: Effects of Copper Addition on Copper Resistance, Antibiotic Resistance Genes, and intl1 during Swine Manure Composting
Source: Front Microbiol. 2017 Mar 3;8:344. doi: 10.3389/fmicb.2017.00344 (PMC5335643; doi:10.3389/fmicb.2017.00344)
Supplement: Supplementary file 1 [file Data_Sheet_1.pdf]

Supporting Information for

Effects of copper addition on copper resistance, antibiotic resistance genes and *intl1*during swine manure composting

Authors: Yanan Yin[1], Jie Gu*[1], Xiaojuan Wang [1], Wen Song[2], Kaiyu Zhang[1], Wei Sun[1], Xin Zhang[2], Yajun Zhang[1], Haichao Li[1]

*Correspondence author: Jie Gu

Affiliation and address:

1. College of Resources and Environmental Sciences, Northwest A & F University, Yangling, Shaanxi 712100, China

2. College of Science, Northwest A&F University, Yangling 712100, China

Email: gujie205@sina.com

Tel: 86-029-87081265

Address: College of Resources and Environmental Sciences, Northwest A & F University, Yangling, Shaanxi 712100, China

Fax: 86-029-87081265

S1. Detailed conditions employed for qPCR

The qPCR reaction mixture comprised 1 μL of DNA template, 0.25 μL of each 0.2 μM primer (ShengGong, China), 10 μL of SuperReal PreMix Plus (TianGen, China), and 8.5 μL of RNase-free water. The qPCR conditions comprised an initial hold for 15 min at 95°C, followed by 40 cycles for 10 s at 95°C, 20 s at the annealing temperatures shown in Table S1, and then 32 s at 72°C. The presence of inhibitory compounds in the extracted DNA was tested by qPCR using serially diluted samples. Melting curve analysis was used to detect nonspecific amplification.

S2. Co-occurrence between CRGs/ARGs subtypes and microbial taxa

Figure 5 contains 35 nodes (28 microbial taxa, five CRGs, two ARGs subtypes, and *intl1*) and 130 edges. Some topological properties that are used widely in network analysis were calculated to describe the complex patterns of the interrelationships among ARG subtypes and microbial taxa. The average weighted degree was 2.528. The average network distance between all pairs of nodes, i.e., the average path length, was 1.49 edges with a network diameter of four edges. The average clustering coefficient was 0.352 and the modularity index was 0.504.

Table S1a. PCR primers

Target genes	Primer Sequences	Amplicon size(bp)	Annealing temp(℃)	(GenBank accession no.)	Reference
erm(A)	TCTAAAAAGCATGTAAAAGAA	641	52	PRJNA352260	[1]
	CTTCGATAGTTTATTAATATTAGT				
erm(B)	GATACCGTTTACGAAATTGG	364	58	X52632	[2]
	GAATCGAGACTTGAGTGTGC				
erm(C)	TCA AAA CAT AAT ATA GAT AAA	642	50	M19652	[2]
	GCT AAT ATT GTT TAA ATC GTC AAT				
erm(T)	CAT ATA AAT GAA ATT TTG AG	369	51	AF310974	[2]
	ACG ATT TGT ATT TAG CAA CC				
erm(X)	GAG ATC GGR CCA GGA AGC	488	58	X51472	[2]
	GTG TGC ACC ATC GCC TGA				
tcrB	CATCACGGTAGCTTTAAGGAGATTTTC	663	55	AY048044.2	[3]
	ATAGAGGACTCCGCCACCATTG				
copA	TGCACCTGACVGGSCAYAT	324	60	M19930<br>CP000075<br>AE016853<br>CP000744<br>EF587902<br>CP000304<br>AE004091<br>AF455751<br>CP000076	[4]
	GVACTTCRCGGAACATRCC				
cusA	ATGCSACVGGYGTTGGCTGG	410	60	CU928162.2	[5]
	CCRTTCAGYTCGGCRATRCC				
pcoA	GCTGCAGATGGCCAGTATGTAAA	147	60	DQ517526.1	[6]
	CCCTCGAGCGTAACCGGTCC				
cueO	ATGGATATGGAACGGATGAA	420	50	HM565232.1	[7]
	ATTGCTACAGATGGTGGTT				
intI1	CTGGATTTCGATCACGGCACG	473	60	DQ393784.1	[8]
	ACATGCGTGTAAATCATCGTCG				
16s rRNA	CCTACGGGAGGCAGCAG	193	55		[9]
	ATTACCGCGGCTGCTGG				

Table S1b. The detailed information of qPCR for each target gene.

**Target gene**	**R^2**	**amplification efficiencies**	**Detection limits (copies/μL)**
copA	0.998	91.9%	22
cusA	0.992	93.3%	28
tcrB	0.999	92.0%	47
pcoA	0.991	106.9%	57
ermA	0.992	92.1%	25
ermB	0.997	91.4%	92
intI1	0.995	99.2%	35
16s rRNA	0.983	92.9%	250

Table S2. Changes in physical–chemical parameters for three treatments

	compost time (days)	pH	NO_3^--N (mg kg^{-1})	NH_4^+-N (mg kg^{-1})	exchangeable Cu (mg kg^{-1})
CK	0	7.85	118.20	1046.00	3.10
	2	8.72	221.69	2187.96	3.64
	7	9.14	188.57	2386.95	3.24
	14	8.38	115.31	1440.44	2.32
	21	8.21	97.80	123.34	1.51
	35	8.51	219.66	201.00	1.49
Cu200	0	7.68	150.31	1159.09	9.70
	2	8.80	234.35	2161.43	9.59
	7	8.88	160.95	1946.85	10.69
	14	8.15	88.75	925.64	4.87
	21	8.25	75.61	275.54	2.85
	35	8.32	81.58	280.00	2.76
Cu2000	0	7.92	158.02	1065.70	112.84
	2	8.61	219.97	3380.73	129.57
	7	9.15	222.33	3302.46	68.67
	14	8.44	125.05	2141.44	49.77
	21	7.99	68.28	49.98	40.89
	35	7.90	51.82	62.00	40.08

Table S3a. Absolute abundance of copper resistance genes and antibiotic resistance*.

		copA	*pcoA*	*cusA*	*tcrB*	*ermB*	*ermA*	*Int1*	16srRNA
	CK	2.6E+08	5.3E+07	8.7E+07	2.4E+07	6.3E+06	3.2E+07	2.5E+08	4.3E+10
		(8.6E+06)	(1.8E+06)	(6.8E+06)	(7.0E+06)	(2.4E+06)	(1.6E+06)	(3.1E+07)	(1.4E+09)
0d	Cu200	1.8E+08	3.2E+07	1.4E+08	3.0E+07	1.7E+07	5.0E+07	4.1E+08	4.2E+10
		(1.3E+07)	(2.6E+06)	(6.6E+06)	(2.0E+06)	(7.3E+06)	(3.3E+06)	(5.7E+07)	(2.4E+09)
	Cu2000	1.4E+08	4.6E+07	1.5E+08	3.0E+07	7.8E+06	4.3E+07	5.4E+08	3.2E+10
		(6.7E+06)	(3.5E+06)	(7.9E+06)	(4.2E+06)	(7.7E+05)	(5.6E+06)	(9.6E+07)	(5.0E+08)
	CK	1.8E+09	5.2E+07	4.0E+08	6.3E+07	1.2E+07	2.7E+07	1.9E+09	5.7E+10
		(9.7E+07)	(4.8E+06)	(2.7E+07)	(1.4E+07)	(7.5E+06)	(1.6E+06)	(5.6E+08)	(3.0E+09)
2d	Cu200	6.0E+08	1.1E+07	2.6E+08	3.7E+07	3.6E+07	2.4E+07	8.5E+08	2.4E+10
		(1.1E+08)	(6.4E+05)	(3.9E+07)	(8.1E+06)	(1.8E+07)	(2.9E+06)	(3.0E+07)	(2.0E+09)
	Cu2000	5.2E+09	2.3E+08	1.1E+09	3.1E+07	3.3E+07	9.1E+07	4.5E+09	9.6E+10
		(3.5E+08)	(2.8E+07)	(7.3E+07)	(1.7E+07)	(6.7E+06)	(2.9E+06)	(7.3E+08)	(4.0E+09)
	CK	9.9E+08	1.2E+08	6.8E+08	4.2E+06	3.1E+07	2.4E+07	1.9E+09	1.9E+11
		(3.1E+08)	(5.1E+07)	(7.9E+07)	(1.7E+06)	(7.5E+06)	(1.5E+06)	(8.1E+07)	(7.5E+09)
7d	Cu200	1.4E+09	1.9E+07	6.7E+08	8.1E+06	1.3E+08	1.2E+07	9.2E+08	1.0E+11
		(1.1E+08)	(7.9E+05)	(9.1E+07)	(3.3E+06)	(1.8E+07)	(1.4E+06)	(1.4E+08)	(7.6E+09)
	Cu2000	1.5E+09	7.7E+06	4.1E+08	6.8E+06	3.3E+07	2.0E+07	1.2E+09	9.6E+10
		(8.1E+08)	(8.2E+04)	(4.9E+07)	(2.9E+06)	(6.7E+06)	(1.6E+06)	(4.5E+07)	(6.5E+08)
	CK	1.3E+09	2.6E+06	4.7E+08	1.1E+06	2.4E+06	4.8E+06	1.7E+08	4.9E+10
		(7.4E+07)	(1.9E+06)	(5.5E+07)	(1.6E+06)	(1.1E+06)	(1.6E+06)	(3.8E+07)	(1.6E+08)
14d	Cu200	2.3E+09	5.8E+06	4.8E+08	9.0E+05	3.1E+06	6.7E+06	2.7E+08	6.3E+10
		(3.2E+08)	(2.7E+06)	(4.1E+07)	(1.7E+05)	(3.1E+05)	(8.4E+05	(1.9E+07)	(5.1E+09)
	Cu2000	8.5E+08	9.8E+06	1.3E+08	3.4E+06	4.4E+06	7.7E+06	3.0E+08	1.5E+10
		(9.9E+07)	(2.1E+06)	(1.7E+07)	(1.6E+06)	(1.1E+06)	(9.2E+05)	(1.1E+07)	(4.8E+08)
	CK	9.2E+08	5.8E+06	8.8E+08	2.0E+06	2.4E+06	1.8E+06	2.5E+08	5.8E+10
		(3.7E+08)	(8.8E+05)	(9.9E+07)	(1.2E+06)	(7.4E+05)	(1.1E+05)	(3.8E+07)	(4.3E+09)
21d	Cu200	3.7E+09	5.6E+05	1.0E+09	8.2E+05	2.5E+06	1.8E+06	1.6E+08	5.9E+10
		(9.2E+08)	(3.0E+05)	(5.3E+07)	(5.1E+05)	(4.0E+05)	(6.7E+04)	(1.9E+07)	(2.1E+09)
	Cu2000	1.7E+09	2.8E+06	8.8E+08	3.9E+06	3.0E+06	9.7E+06	4.0E+08	6.1E+10
		(9.9E+07)	(9.1E+05)	(7.1E+06)	(1.3E+06)	(2.5E+05)	(1.4E+06)	(1.1E+07)	(1.1E+09)
	CK	1.9E+09	1.6E+07	9.5E+08	9.7E+05	2.2E+06	1.7E+06	2.1E+08	6.1E+10
		(1.6E+08)	(1.2E+07)	(6.9E+07)	(3.7E+05)	(3.5E+05)	(2.0E+05)	(1.6E+07)	(2.8E+09)
35d	Cu200	3.4E+09	1.6E+06	5.1E+08	3.7E+05	1.3E+06	1.2E+06	1.5E+08	3.2E+10
		(5.5E+08)	(6.2E+05)	(2.2E+07)	(5.3E+04)	(3.6E+05)	(1.7E+05)	(1.5E+07)	(1.1E+09)
	Cu2000	4.2E+09	1.7E+04	8.5E+08	2.9E+06	8.4E+05	3.0E+06	2.2E+08	3.9E+10
		(1.4E+09)	(4.6E+03)	(9.0E+07)	(1.5E+06)	(8.3E+03)	(2.6E+05)	(1.2E+07)	(7.1E+08)

*mean ± (S.E.)

Table S3b. The dynamic changes of log transformed gene copies during swine manure composting

Target genes	Control						Cu200						Cu2000					
	D0	D2	D7	D14	D21	D35	D0	D2	D7	D14	D21	D35	D0	D2	D7	D14	D21	D35
copA	8.4	9.3	9.0	9.1	9.0	9.3	8.3	8.8	9.1	9.4	9.6	9.5	8.1	9.7	9.2	8.9	9.2	9.6
pcoA	7.7	7.7	8.1	6.4	6.8	7.2	7.5	7.0	7.3	6.8	5.7	6.2	7.7	8.4	6.9	7.0	6.4	4.2
cusA	7.9	8.6	8.8	8.7	8.9	9.0	8.1	8.4	8.8	8.7	9.0	8.7	8.2	9.0	8.6	8.1	8.9	8.9
tcrB	7.4	7.8	6.6	6.1	6.3	6.0	7.5	7.6	6.9	6.0	5.9	5.6	7.5	7.5	6.8	6.5	6.6	6.5
ermA	7.5	7.4	7.4	6.7	6.3	6.2	7.7	7.4	7.1	6.8	6.2	6.1	7.6	8.0	7.3	6.9	7.0	6.5
ermB	6.8	7.1	7.5	6.4	6.4	6.3	7.2	7.6	8.1	6.5	6.4	6.1	6.9	7.5	7.5	6.6	6.5	5.9
intI1	8.4	9.3	9.3	8.2	8.4	8.3	8.6	8.9	9.0	8.4	8.2	8.2	8.7	9.7	9.1	8.5	8.6	8.3

Table S4. The Spearman's correlation analysis between relative abundances of CRGs, ARGs, *intl1* and exchangeable Cu during the swine manure composting

	copA	*CusA*	*pcoA*	*tcrB*	*emA*	*emB*	*Intl1*	Cu
copA	1	0.723**	-0.388**	-0.411**	-0.365**	-0.303*	-0.139	-0.031
CusA		1	-0.590**	-0.408**	-0.580**	-0.463**	-0.352**	-0.245
pcoA			1	0.572**	0.724**	0.516**	0.670**	0.326*
tcrB				1	0.747**	0.492**	0.639**	0.399**
emA					1	0.645**	0.787**	0.662**
emB						1	0.721**	0.535**
Intl1							1	0.716**
Cu								1

* Correlation significant at $p < 0.05$.

** Correlation significant at $p < 0.01$.

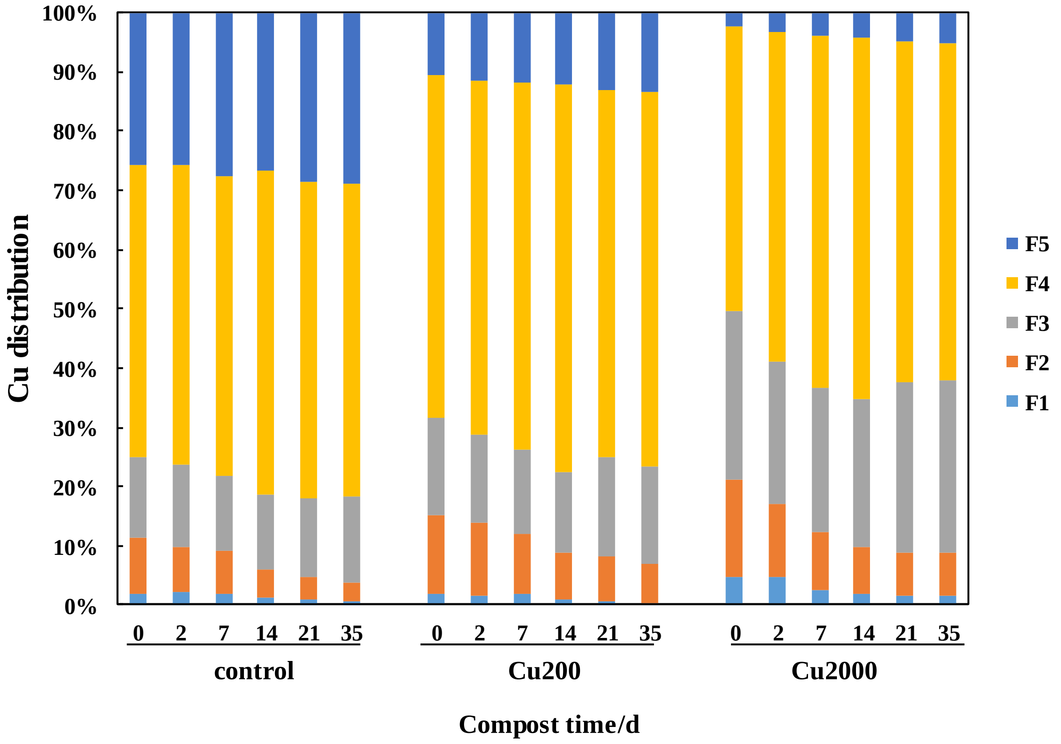

Figure S1. Changes in Cu speciation during manure composting of control (A), Cu200 (B) and Cu2000(C), respectively.

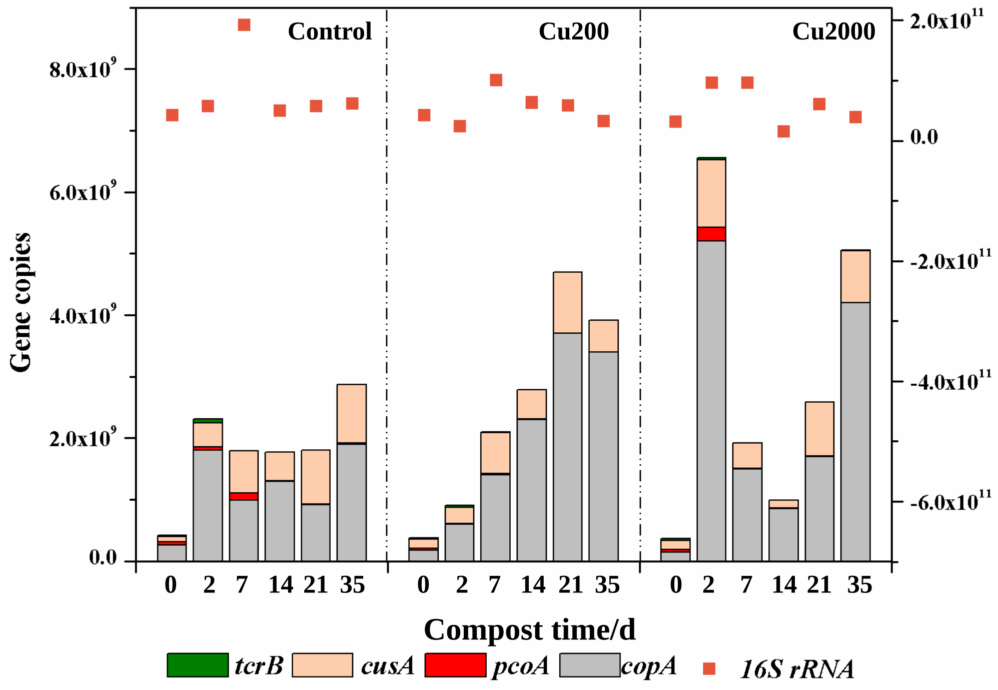

Figure S2. Changes of absolute abundance CRGs and 16S rRNA gene during manure composting of control (A), Cu200 (B) and Cu2000(C), respectively.

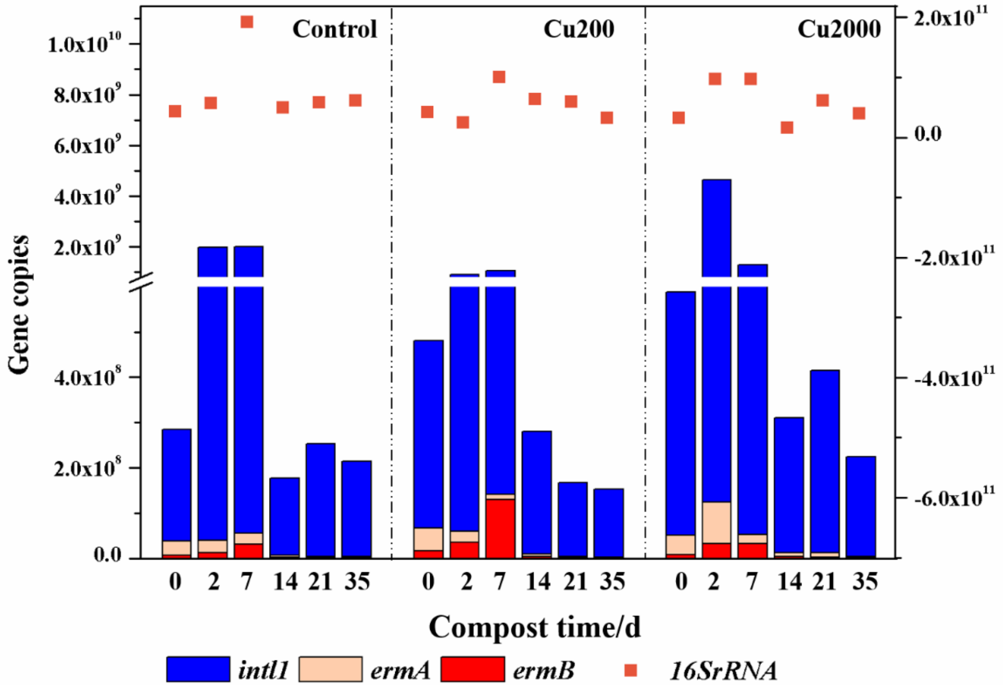

Figure S3. Changes of absolute abundance ARGs and *intI1* gene during manure composting of control (A), Cu200 (B) and Cu2000 (C), respectively.

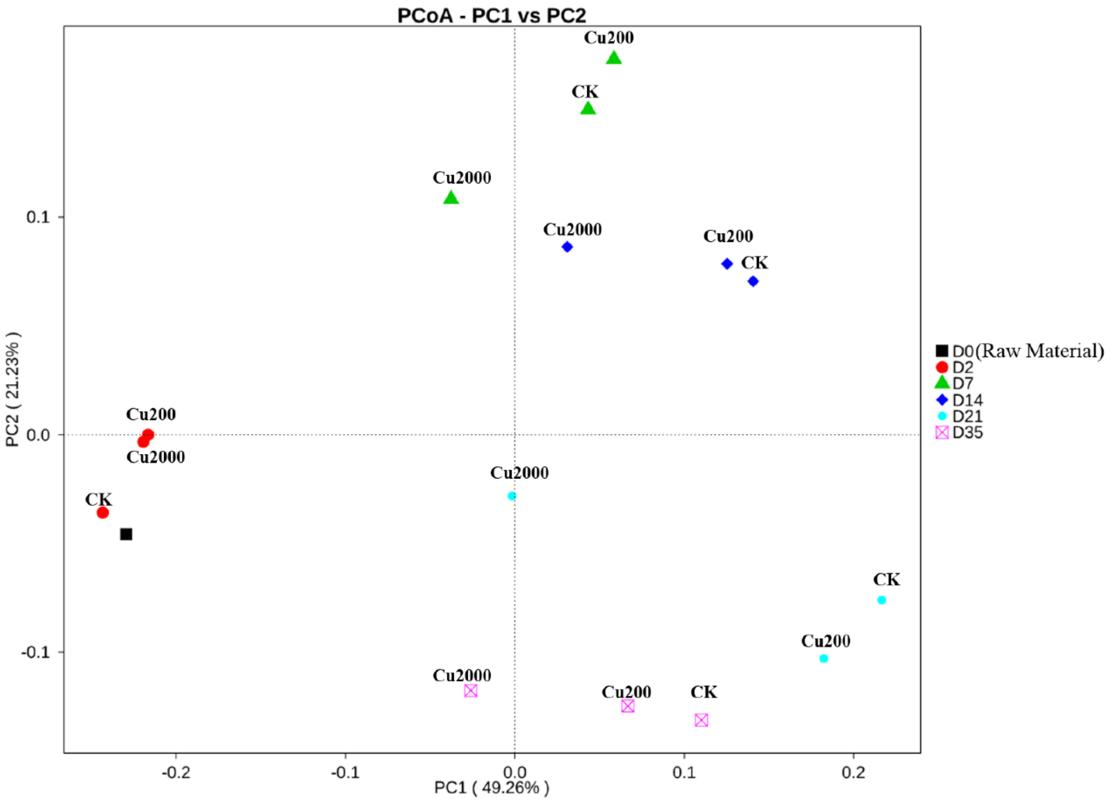

Figure S4. Principal coordinate analysis of the bacterial communities during manure composting of control (A), Cu200 (B) and Cu2000 (C), respectively.

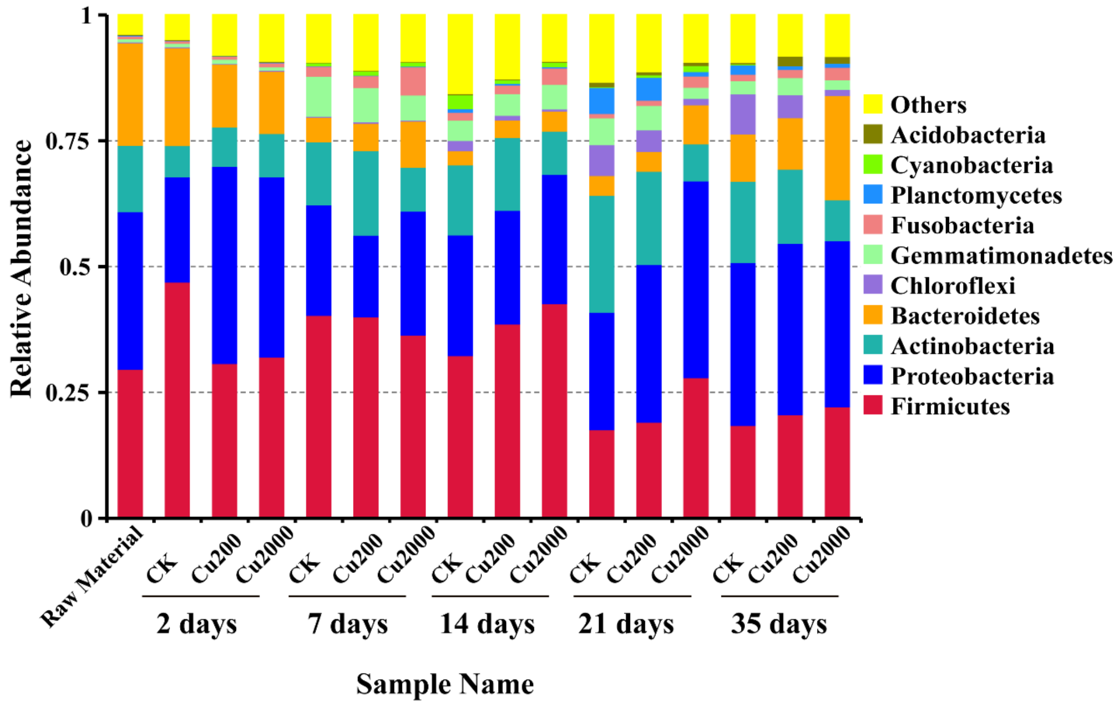

Figure S5. Variations in the main phyla detected during manure composting of control (A), Cu200 (B) and Cu2000 (C), respectively.

References:

[1] Chung, W.O., Werckenthin, C., Schwarz, S., Roberts, M.C., 1999. Host range of the ermF rRNA methylase gene in bacteria of human and animal origin. J.Antimicrob. Chemoth. 43, 5−14.

[2] Chen, J., Yu, Z., Jr, M.F., Wittum, T., Morrison, M., 2007.Development and application of real-time PCR assays for quantification of erm genes conferring resistance to macrolides-lincosamides-streptogramin B in livestock manure and manure management systems. Appl. Environ. Microbiol, 73, 4407−4416.

[3] Hasman, H., Kempf, I., Chidaine, B., Cariolet, R., Ersbøll, A.K., Houe, H., Bruun Hansen, H.C., Aarestrup, F.M., 2006. Copper resistance in Enterococcus faecium, mediated by the tcrB gene, is selected by supplementation of pig feed with copper sulfate. Appl. Environ. Microbiol. 72, 5784−5789.

[4]Li, X.F., Yin, H.B., Su, J.Q., 2012. An Attempt to Quantify Cu-Resistant Microorganisms in a Paddy Soil from Jiaxing, China. Pedosphere. 22, 201−205.

[5] Besaury, L., Bodilis, J., Delgas, F., Andrade, S., Iglesia, R.D.L., Ouddane, B., Quillet, L., 2013. Abundance and diversity of copper resistance genes cus A and cop A in microbial communities in relation to the impact of copper on Chilean marine sediments. Mar. Pollut. Bull. 67, 16−25.

[6] Xiong, W., Zeng, Z., Zhang, Y., Ding, X., Sun, Y., 2015. Fate of metal resistance genes in arable soil after manure application in a microcosm study. Ecotoxicol.

Environ. Saf. 113, 59−63.

[7]Zhang J, Chen M, Sui Q, et al. Impacts of addition of natural zeolite or a nitrification inhibitor on antibiotic resistance genes during sludge composting.[J]. Water Research, 2016, 91(9):1286-91.

[8] Stokes, H. W., Nesbø, C. L., Holley, M., Bahl, M. I., Gillings, M. R., Boucher, Y., 2006. Class 1 integrons potentially predating the association with tn402-like transposition genes are present in a sediment microbial community. J. Bacteriol. 188, 5722−5730.

[9]Aminov, R. I., Chee-Sanford, J. C., Garrigues, N., Teferedegne, B., Krapac, I. J., White, B. A., Mackie, R. I. Development, validation, and application of PCR primers for detection of tetracycline efflux genes of gram-negative bacteria. Appl. Environ. Microbiol. 68, 1786−1793.
